# Ten Simple Rules for a Bioinformatics Journal Club

**DOI:** 10.1371/journal.pcbi.1004526

**Published:** 2016-01-28

**Authors:** Andrew Lonsdale, Jocelyn Sietsma Penington, Timothy Rice, Michael Walker, Harriet Dashnow

**Affiliations:** 1 ARC Centre of Excellence in Plant Cell Walls, School of BioSciences, University of Melbourne, Parkville, Victoria, Australia; 2 Bioinformatics, Walter and Eliza Hall Institute of Medical Research, Parkville, Victoria, Australia; 3 School of Mathematics and Statistics, University of Melbourne, Parkville, Victoria, Australia; 4 School of BioSciences, University of Melbourne, Parkville, Victoria, Australia; 5 Bioinformatics, Murdoch Childrens Research Institute, Parkville, Victoria, Australia; 6 Life Science Computation Centre, Victorian Life Sciences Computation Initiative, Carlton, Victoria, Australia; Whitehead Institute, UNITED STATES

## Introduction

As science becomes increasingly interdisciplinary, we are expected to acquire both breadth of knowledge and depth of expertise. In bioinformatics, this is especially true. Keeping up to date with major techniques across multiple specialisations can be daunting, but you need not face it alone.

A journal club is an excellent way to take in the scientific literature, keep up with developments in your field, and hone your communication and analytical skills.

In general, a journal club is a group of people who meet regularly to discuss one or more scientific papers. The structure of such a club can vary. In the more traditional format, an individual studies a paper and then presents it to the group, usually in the form of PowerPoint slides, with time for questions. In some institutions, the journal club is for students only, designed to fulfill the requirements of a course or postgraduate program; attendance is obligatory, the scope of the literature is narrow, and the format is prescribed. The preparation of slides and a lecture may be required. Other kinds of journal clubs are just lab meetings in disguise, with the usual lab head and group members in attendance and one member nominated to present the paper.

A formal style often fits well within an established academic structure—but what if your discipline is emerging?

Consider the field of bioinformatics. Expertise may be spread across departments and institutions, and there may not be an obvious place or critical mass in any one lab for a traditional journal club. Research students, “pet bioinformaticians,” [1] and those interested in bioinformatics from adjoining fields all need a place to gather.

We are pleased to offer an alternative structure to address this situation—an informal journal club, designed to bring together a diversity of backgrounds and career stages to discuss bioinformatics while building a network of like-minded peers. Additional benefits of such a journal club may include friendship and breakfast (see Rule 2)! We thoroughly recommend it to anyone who asks (as well as those who don’t).

While this advice is drawn from our experiences in the Parkville Bioinformatics Journal Club, it is applicable to developing informal journal clubs of all disciplines. The advice contained in these rules will also help those who want to spice up their existing formal format.

So don’t be a “lonely bioinformatician”[1], create a journal club! Follow these Ten Simple Rules to find out how.

## Rule 1: Have It at 8 a.m.

**Fig 1:**
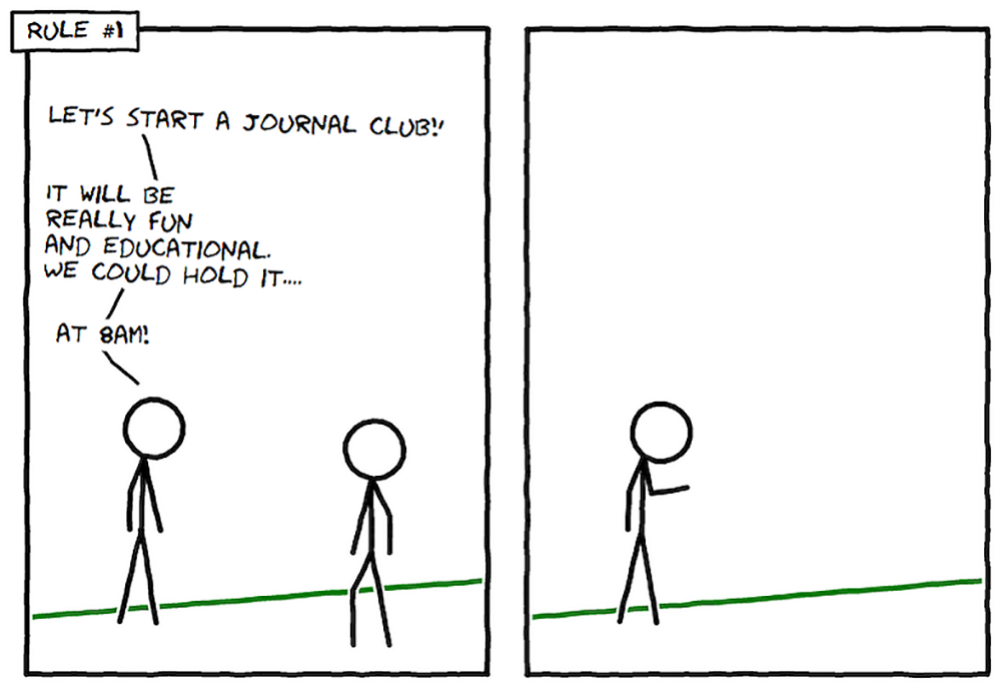
Have it at 8 a.m.! This comic was drawn using Comix I/O (http://cmx.io/).

Our research has indicated that the best time to hold a journal club is at 8 a.m. (*n* = 1). The most common reaction to this conclusion is best summarised in Fig 1. Words like “lunchtime,” “evening,” and “weekend” are likely to elicit similar reactions and, along with 8 a.m., seem unlikely times for a journal club. However, there is a method to our madness.

Forming an interdisciplinary journal club typically requires volunteers from various areas with competing schedules and commitments. This makes finding a suitable time challenging. We recommend you find a time that is typically free for the majority and suits both students and early career researchers, who may have time commitments during “normal” working hours. For us, that time was 8 a.m.

We chose our meeting time by polling members of our bioinformatics community. Any club consists primarily of its members and requires a culture of willing participation to run well. Mere attendance will not do the job. A time outside regular commitments may appear difficult at first, but attendance indicates that members truly wish to participate; otherwise, they would not make the effort to appear at all!

The caveat to this is that this noncompulsory attendance results in only people of a certain disposition attending; in this case, those for whom an 8 a.m. optional journal club is appealing. This is a self-selection bias that we can live with.

An early start means that members are neither tired from nor distracted by the regular issues and chores of the day. There was also the bonus that it meant that members were on campus early in the morning at least once a fortnight—a vast improvement for some of the authors.

If those interested in starting a journal club are early risers, then follow our lead, but be sure to meet the needs of your own community. In setting up an unconventional journal club, there is scope for unconventional structure.

## Rule 2: Keep It Casual (with Coffee!)

**Fig 2:**
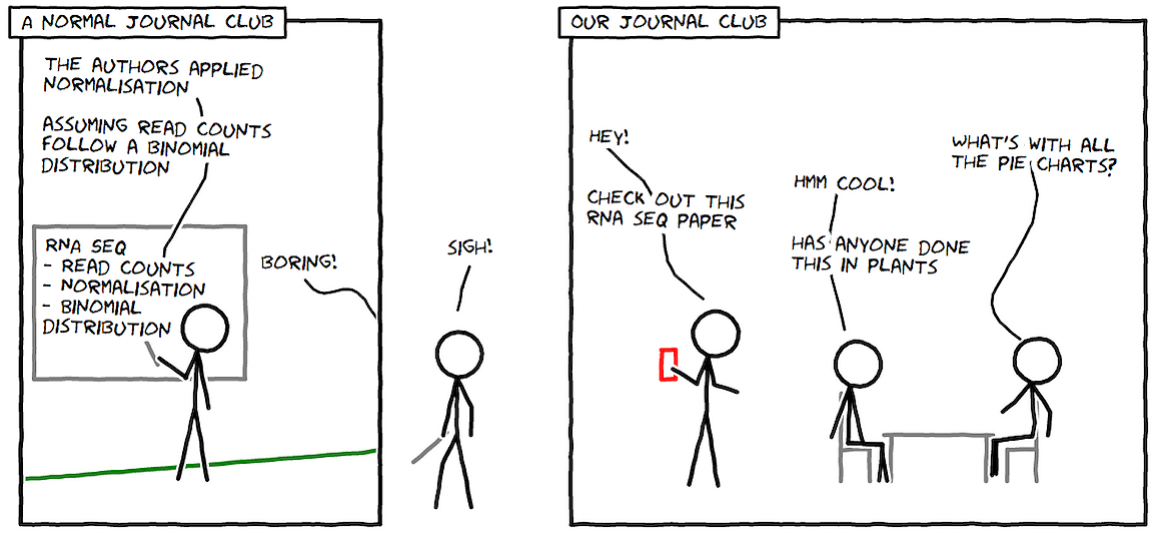
Why our journal club beats a “normal” journal club. This comic was drawn using Comix I/O (http://cmx.io/).

Having decided when you will hold your journal club, a location is the next requirement. For an 8 a.m. start (Rule 1), the priority for the caffeine-fueled organisers was finding a venue with great coffee. This initial choice of venue helped form additional requirements. Adding a journal club to already busy schedules can require an incentive. For our members, breakfast and coffee made the early start bearable, so a venue with a variety of food and drink, including coffee, was essential. Available tables (without the overhead of booking) and proximity to the campus precinct were also desirable traits. This placed the club in a cafe, which also ruled out formal presentations with PowerPoint slides (much to our relief), and this choice of location shaped our journal club (Fig 2).

The choice of an 8 a.m. start helped form the mood and style of our journal club, and the time that suits your community will do the same.

If you are holding your journal club in a meeting room, consider some other kind of incentive to make your club more appealing and encourage people along. For example, a rotation of baking or bringing along snacks. If it is at lunchtime, include lunch as part of the schedule. Since the club is (ideally) relaxed and social, it relies on its members being genuinely enthusiastic in order to function, so a style that suits your community is essential.

Over the course of journal club meetings, our style quickly developed. An informal location with food or drink lends a welcoming air. The early start and ability to order food and drink also meant that 8.10 a.m. became the de facto start time, as members tended to arrive on time and then place an order; this set the relaxed tone that followed. The leader of the session gives a short overview of the paper, explains what drew them to choose it, and then opens it up for discussion. That is as much structure as we needed.

This style makes it easy for new people to attend. They can just sit and listen until they feel comfortable enough to contribute. Latecomers are not admonished, and on more than one occasion, participants admitted to having not read all (or any) of the selected papers! This kind of admission is unlikely to be well received in a formal club but is met only with gentle teasing in ours.

Where you decide to hold your journal club will affect the style and format, so ensure your venue matches the type of club you are aiming for.

## Rule 3: Multidisciplinary Is Not a Dirty Word

As we’ve alluded, the traditional journal club format often isn’t a good fit for bioinformatics. Our members come from various universities and institutes, many from groups in which they are the “lone bioinformaticians” or where all their lab mates are computational and they are just learning to program. They feel alone in their own labs, so they come to a journal club to feel part of a larger field.

This group may call themselves many different things (e.g., bioinformaticians, computational biologists, systems biologists) and have different areas of expertise. We have molecular modelers, computer scientists, mathematicians, and cancer genomics gurus. Add to this mix biologists seeking more bioinformatics knowledge, and the range of expertise is even broader.

This multidisciplinary context is a challenge, but it is this diversity that is part of what makes our journal club so special.

It can be intimidating to come into a group where everyone knows each other or everyone is expert on the same topic and you are not. The breadth of expertise is an advantage in this case; our diverse group means that for every paper, there will be someone who knows nothing about the topic and others who are near-experts.

By having an unconventional approach and an informal style, we create a safe environment where everyone feels comfortable asking questions when they don’t understand, and others flex their teaching skills by answering these questions. With so many people coming to new fields of study with their own unique backgrounds, we inevitably discuss harebrained solutions to seemingly insurmountable problems. We encourage questions, opinions, and tangents. Ensure the group is diverse by inviting people from a range of labs and departments. We have an open membership approach and encourage attendance from different cohorts. Mixing research students, coursework students, and early career researchers helps give perspectives from differing stages of career and life!

A mixed group can prevent situations where complex methods might be skipped because they are outside the expertise of the group, as what may appear to be complex to newcomers can often be explained succinctly by those familiar with it.

By having a diverse group of people in which someone is almost always bound to know an answer, you create an environment that is valuable to someone trying to learn about other disciplines. Many of us involved in bioinformatics were at one stage computer scientists without a biology background or biologists without computing or statistics. Expertise in everything is unlikely, and there will inevitably be gaps.

The diversity of a journal club is a way to fill in those gaps and benefit from our shared knowledge.

## Rule 4: Let Your Topics Be As Diverse As Your Members

With a regular time and venue arranged, the topics to be covered in your journal club need to be defined.

Though some journal club formats have a narrow scope of topics, restricted to the research focus of a lab or from a prescribed list, there are benefits to having a more relaxed approach. Given the diverse backgrounds of our members (see Rule 3), and our informal approach, we do not prescribe topics.

Members are free to volunteer to lead on a topic of their choosing. This flexibility allows for a range of discussion styles—teaching from experience in one’s own field, branching out into something new to everyone, or even using the journal club as a crowd-sourcing opportunity for a critical paper in your literature review.

This flexibility has led to a broad range of topics. Scientific topics have included synthetic biology, glycoinformatics, ChIP-seq, the human microbiome, systems biology, CRISPR-Cas9, and molecular modelling.

A variety of topics can also act as good entry points to your club. A constant schedule of scientific topics may intimidate new entrants to the field or those who are curious about your club. The occasional topic that focuses on general skills for science, or issues common to all disciplines, can encourage new members and reinforce that your club is atypical.

We have discussed overcoming procrastination, writing more effectively, online learning, editing Wikipedia, student groups, Software Carpentry, document preparation systems for science, and even the PLOS Ten Simple Rules collection itself!

Leaders are free to choose areas of research that they know well and will enjoy dissecting but need to remember that the rest of the group may not share their expertise or personal interest. It is often helpful to accompany domain-specific articles with a suitable review paper to introduce others to the important concepts.

Frequently study papers that are neither fashionable nor trending. The major breakthroughs of tomorrow are among the peripheral topics of today, and the mainstream is, almost by definition, slow to recognise the truly revolutionary. This was recognised by Baez [2]: “Look for problems, not within disciplines, but in the gaps between existing disciplines. The division of knowledge into disciplines is somewhat arbitrary, and people put most of their energy into questions that lie squarely within disciplines, so it shouldn’t be surprising that many interesting things are lurking in the gaps, waiting to be discovered.”(Aside: this blog also made for a good journal club meeting.)

Even papers destined for obscurity provide a new perspective, and some of our most stimulating discussions were based on papers from adjoining fields such as genetics and information theory.

## Rule 5: Find Good Articles For Discussion

A high-quality journal article doesn’t necessarily make for a good journal club topic. It’s important to read good science, but journal club is also about honing your analytical skills. Find papers you respect enough to criticise. Look for papers with interesting ideas, issues, or challenges that you can discuss.

The articles you choose should be specific enough to gain interest, yet flexible enough to engage the diverse members of the group. It is good to choose a challenging article, but not too challenging. If the paper is dense, it should also be short. Remember that your members are most likely reading this article in their spare time.

For the same reasons, avoid big papers: it is hard to digest and critique ten years’ worth of work over one coffee (or even two).

How do you find topical journal articles? RSS feeds can be used to suggest relevant articles, as can reviews. When choosing articles, accessibility can be an issue, particularly if you have members who don’t have access to big university libraries. Consider choosing articles from open access journals.

In an interdisciplinary club, members will have various backgrounds. Consider additional resources for varying levels of expertise. A selection of the current literature could optionally be accompanied by links to seminal papers or online primers such as Wikipedia to give context.

**Fig 3:**
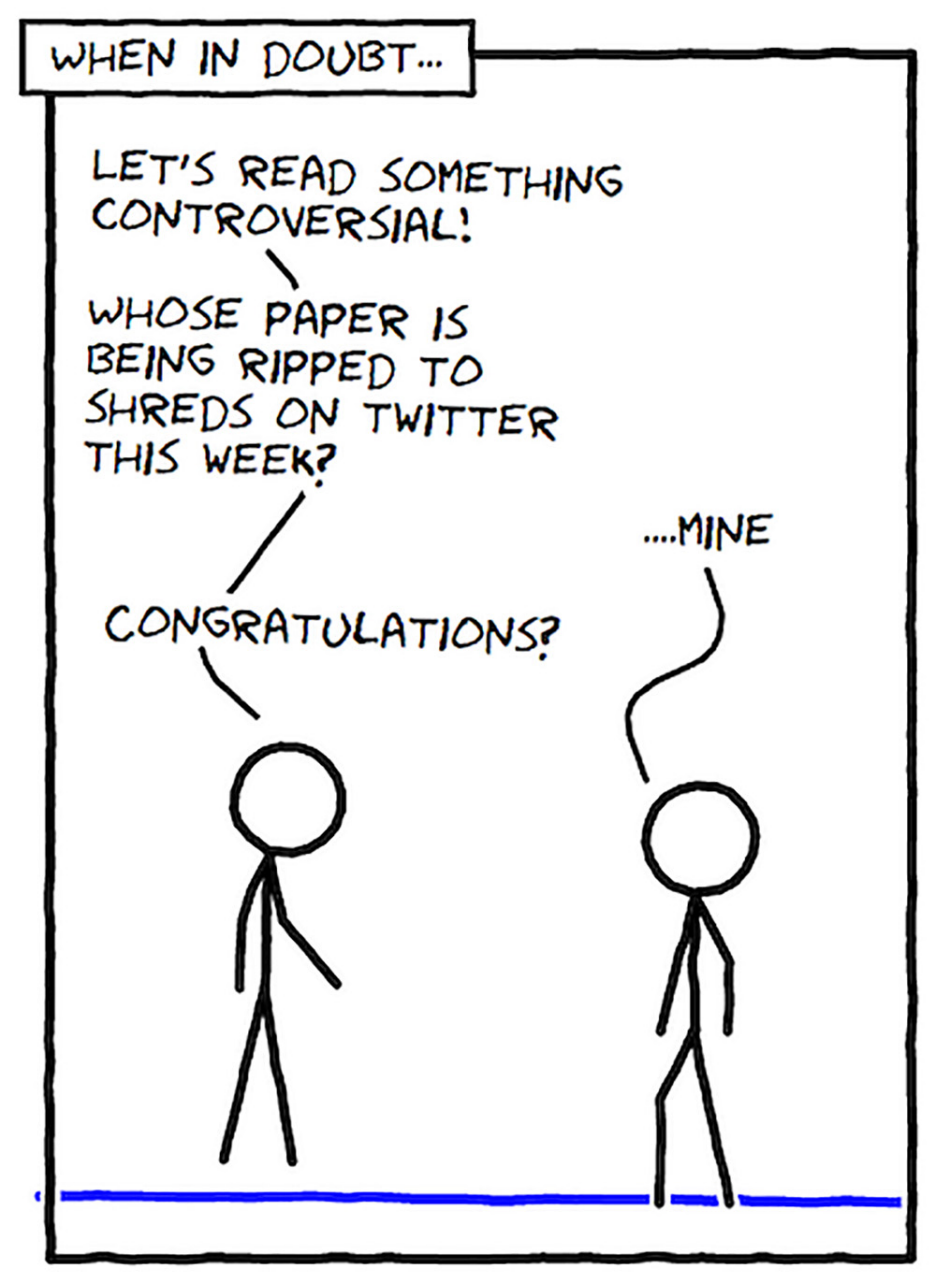
How to find contentious articles. This comic was drawn using Comix I/O (http://cmx.io/).

Papers don’t have to be on paper. Blogs, social media, and other online resources can add variety to your discussion diet. Twitter and blogs can be particularly useful if you’re looking for something topical or contentious (Fig 3). Some blogs have a certain renown for applying strong criticism to published journal articles. It can make for lively conversation to first read an article that seems like valid work from leading people in a field, and then read a blog post that tears the same article to shreds.

Social media, post-publication peer review, preprints, and open access are changing the way we communicate scholarly information. Let them change the way we select articles for journal clubs as well.

## Rule 6: Make Leading Easy

As people join your journal club, expanding the roster of leaders is important. An informal club relies on volunteers, so making it easy to lead a session is critical. Founders of the club may lead more often than others, but a club works best when everyone shares the article selection and leadership.

Moving from participating to leading can be daunting. It need not be. We keep the format simple and provide a quick-start guide to get new members up to speed. A short document or a blog post (such as this one we prepared earlier: http://parkville-bioinformatics-journal-club.blogspot.com.au/2014/01/leading-journal-club_28.html) with advice on how your club runs is a simple way to impart advice. You can also use this document to elaborate on the benefits of leading a journal club session.

Each journal club leader brings their own personality to the task. One memorable opening line, “Welcome to Journal Club,” remains the best and only formal start to a journal club so far, but it all depends on the style. In general, introduce yourself, and ask for a brief round of introductions. A simple introduction at the start can make newcomers more relaxed about joining in, and help those with failing memory remember names.

One benefit of leading journal club is you can use it to forward your other academic goals. The discussion at journal club could provide motivation and fresh perspectives for future projects, and any material prepared for the discsussion can be fodder for your next literature review, journal article, or blog post.

## Rule 7: Avoid Dead Air

Like good radio, a journal club works best when there is constant sound. Newcomers should not feel pressured to participate prematurely, but try to keep the conversation going when those inevitable lulls occur. This is primarily the leader’s responsibility, and it helps to bring a list of questions or comments.

Give a brief summary of what the paper was about. Think of this as a verbal abstract, but with even less detail. The idea is to give people who haven’t read it (or have only skimmed it) a basic idea of what the paper was about. You may want to finish with a comment about what you thought of the paper. What were its strengths and weaknesses? Why did you choose it?

However, the leader is not alone. All members should feel able to chip in when there is a gap, and like commercial radio, there is nothing wrong with the odd cliché, e.g., “What are the next steps from this paper?”, or as one of the authors (AL) asks almost every week, “Has this been done in plants?”

Avoiding an awkward silence need not be a chore. In an informal journal club, going off topic is encouraged! Feeling comfortable enough to ask questions that you would not ask in a more formal setting is a sign that your journal club has the right spirit.

## Rule 8: Be Organised

A journal club of only one person is referred to as “reading alone.” Success depends on people who turn up consistently and volunteer to select papers, so it helps to stay organised.

Communicate regularly with email, Facebook, or cans and bits of string—whatever works, or use them all! What is the best way to distribute papers? Google Drive, or email PDFs? Emailing links has sometimes been fraught with problems. Sorry, we don’t know the optimal answer for this one!

Ongoing recruitment and encouragement is important—otherwise, journal club will become a shrinking clique. Advertisements, Facebook, and reminder emails all have a role, and remember to make it easy for new leaders (see Rule 6).

Informal does not mean improvised. Schedule leaders in advance and announce articles to give time for everyone to read them. Always know when the next meeting is and who is responsible for choosing a paper. For example, our club found that after the end of year break, holding a meeting at least symbolically in January (summer break in the southern hemisphere) ensured that the club was able to continue momentum into the new year.

**Fig 4:**
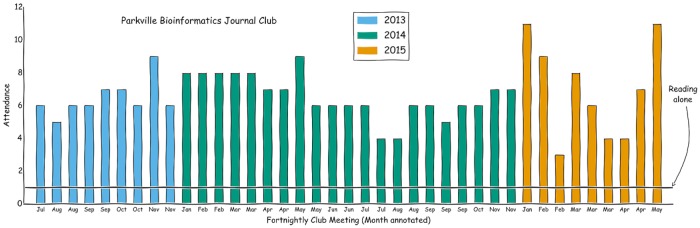
Journal club attendance over time versus reading alone.

Keeping track of the next meeting means you need to keep records. When are the meetings? Who led, how many attended, and which papers were discussed? This is useful not only for fancy graphs (see Fig 4) but also for letting newcomers know about the history of the club and which topics have already been covered. It also means that you can check your facts and find citations if you need to write a Ten Simple Rules for a Bioinformatics Journal Club.

## Rule 9: Be Adaptable

One particular strength of an informal journal club is its ability to adapt to the needs of its members.

For example, when scheduling your journal club, be sure to consider all of the factors that impact your members, such as holidays, exams, PhD confirmations, and grant writing. If necessary, reduce the frequency of meetings at such times.

It’s easiest to organise around weekly meetings but hard to arrange a different leader and read so many papers every week. If, like us, you find a fortnightly meeting easier to sustain, then it is useful to have something else in the intermediate weeks. Our club has a writing gym (“pomodoro” sessions) modelled after Shut up and Write! (http://thesiswhisperer.com/shut-up-and-write/) on the alternate weeks, but yours might have different needs. Having that timeslot reserved for your group every week will help people trust that they can always show up at that time and catch up with colleagues over a coffee.

Although we didn’t originally envisage journal club as a platform for collaborations, we’ve found that meeting regularly to write amongst a group of supportive peers has led to a publication, with a style that suits the informal atmosphere of the club [3].

Viewing the club as more than just a journal club has been an invaluable chance for exposure as early career researchers. An earlier version of these rules appeared as a poster at the inaugural Australian Bioinformatics Conference (http://bioinformatics.net.au/abic2014/) and is available at F1000Research (http://f1000.com/posters/browse/summary/1097136).

It turns out that journal club can be more than just reading papers, which leads us to Rule 10.

## Rule 10: It’s about More than Just the Articles

The encouragement to read and the opportunity to discuss are why we meet, but the biggest gain is actually a community of peers.

The most exciting bioinformatics journal clubs consist of members with diverse backgrounds, acting as a microcosm for the field of bioinformatics at large. Our journal club members come from all over the field of bioinformatics.

A diverse background of journal club members makes for a diverse selection of literature, acting as revision for some and revelation for others. The articles we discuss not only improve our understanding of bioinformatics but also broaden our vision of what bioinformatics contains.

Drawing together a heterogeneous group of students and early career researchers on a regular basis creates a network that spans the existing structures of labs, departments, faculties, and schools. A vibrant journal club becomes something more than the sum of its parts. It becomes a community.

Coffee, articles, friendship, news, research, support, and ideas merge. You can measure the success of a journal club by more than just how many attend, or the impact factor of the articles selected. Your journal club will be successful when you don’t even think of it as a club at all.

## Conclusions

Remember to enjoy yourself. In an unconventional journal club, you are not giving a formal presentation. No one is judging you, but if they are, give them a copy of these Ten Simple Rules.

Create an informal atmosphere for your journal club. We do this using coffee, but you need to find what works for your community. You may wish to complement your journal club with other activities such as a writing club or social events.

Choose a venue that matches the intended style of your club. Invite and encourage a diversity of backgrounds and expertise to your journal club, and let the topics and articles reflect that diversity.

Keep the barrier to entry low. Because attendance is not compulsory, journal clubs like this rely on volunteers to choose the papers and lead the session. It needs to be easy for people to do this. There should be simple and easy-to-follow guidelines to help members move from observers to active participants (see Rule 6).

Reading bad papers can be good. Whereas your supervisor may tend to focus on fairly specific and conventional literature, a journal club is a chance to discuss articles that may be controversial or even flawed. Don’t be afraid to look outside your field for a fresh perspective. We also recommend supplementing journal articles with less formal commentary such as blogs.

Finally, we would like to leave you with this thought: journal club is not really about reading and discussing journal articles. Sure, that’s what we drag ourselves out of bed to do at 8 a.m. on a Tuesday morning, but the true purpose of journal club is to build a bioinformatics community that spans institutes, ages, and even disciplines.

Journal club is about more than just the articles; it’s about the people you share them with.
